# Gossypol induces apoptosis in multiple myeloma cells by inhibition of interleukin-6 signaling and Bcl-2/Mcl-1 pathway

**DOI:** 10.3892/ijo.2014.2652

**Published:** 2014-09-15

**Authors:** KEN SADAHIRA, MORIHIKO SAGAWA, TOMONORI NAKAZATO, HIDEO UCHIDA, YASUO IKEDA, SHINICHIRO OKAMOTO, HIDEAKI NAKAJIMA, MASAHIRO KIZAKI

**Affiliations:** 1 Division of Hematology, Department of Internal Medicine, Keio University School of Medicine, Tokyo 160-8582, Japan; 2 Department of Hematology, Saitama Medical Center, Saitama Medical University, Saitama 350-8550, Japan; 3 Department of Hematology, Yokohama Municipal Hospital, Kanagawa 240-8555, Japan; 4 Department of Laboratory Medicine, Tokyo Electric Power Company Hospital, Tokyo 160-0016, Japan

**Keywords:** multiple myeloma, gossypol, apoptosis, bcl-2, interleukin-6

## Abstract

Multiple myeloma (MM) is a clonal plasma cell disorder affecting the immune system with various systemic symptoms. MM remains incurable even with high dose chemotherapy using conventional drugs, thus necessitating development of novel therapeutic strategies. Gossypol (Gos) is a natural polyphenolic compound extracted from cotton plants, and has been shown to possess anti-neoplastic activity against various tumors. Recent studies have shown that Gos is an inhibitor for Bcl-2 or Bcl-X_L_ acting as BH3 mimetics that interfere interaction between pro-apoptotic BH3-only proteins and Bcl-2/Bcl-X_L_. Since most of the patients with MM overexpress Bcl-2 protein, we considered Gos might be a promising therapeutic agent for MM. We herein show that Gos efficiently induced apoptosis and inhibited proliferation of the OPM2 MM cell line, in a dose- and time-dependent manner. Gos induced activation of caspase-3 and cytochrome *c* release from mitochondria, showing mitochondrial dysfunction pathway is operational during apoptosis. Further investigation revealed that phosphorylation of Bcl-2 at serine-70 was attenuated by Gos treatment, while protein levels were not affected. In addition, Mcl-1 was downregulated by Gos. Interestingly, phosphorylation of JAK2, STAT3, ERK1/2 and p38MAPK was inhibited by Gos-treatment, indicating that Gos globally suppressed interleukin-6 (IL-6) signals. Moreover, JAK2 inhibition mimicked the effect of Gos in OPM2 cells including Bcl-2 dephosphorylation and Mcl-1 downregulation. These results demonstrated that Gos induces apoptosis in MM cells not only through displacing BH3-only proteins from Bcl-2, but also through inhibiting IL-6 signaling, which leads to Bcl-2 dephosphorylation and Mcl-1 downregulation.

## Introduction

Multiple myeloma (MM) is a clonal plasma cell disorder affecting both the immune system and bone metabolism, and it remains incurable even with high dose chemotherapy (1,2). Chemoresistance of MM could be, in part, attributed to the overexpression of anti-apoptotic protein, BCL-2 (3–5). In fact, >80% of the patients with MM overexpress BCL-2 protein, and increased level of BCL-2 is associated with poor overall survival (6).

Human *BCL-2* gene is located on chromosome 18, at the breakpoint of t(14;18), a chromosomal translocation that was first discovered in follicular lymphoma (7,8). This translocation juxtaposes BCL-2 gene to the enhancer of immunoglobulin heavy chain gene, resulting in the overexpression of BCL-2 protein. Notably, overexpression of BCL-2 is not only observed in tumors with t(14;18), but frequently seen in a variety of hematological malignancies without t(14;18) (9,10). Increased BCL-2 protein in tumor cells stabilizes mitochondrial membrane and prevents the release of cytochrome *c* from mitochondria, consequently interrupting the intrinsic apoptotic signaling cascade (11–13). Therefore, downregulation of BCL-2 protein can restore intrinsic apoptotic pathways and resensitize tumor cells to apoptosis (10). In fact, current therapeutic approaches to boost apoptosis in malignant cells often target BCL-2 family members, and such approach is particularly effective for tumors overexpressing Bcl-2 including MM (14).

Gossypol (Gos) is a promising anticancer agent presently under clinical trial (15). Gos is a natural polyphenol compound extracted from cottonseeds (16), which was initially investigated in China as a male contraceptive agent (17). Gos is a natural BH3 mimetics, acting as a small molecule inhibitor for the interaction between anti-apoptotic Bcl-2/Bcl-X_L_/Mcl-1 and pro-apoptotic BH3-only proteins such as BIM, BID, BAD or BIK, thereby induces apoptosis in cancer cells (18). Recent observations indicated antiproliferative or antimetastatic activity of Gos in several tumor types, including human breast carcinoma, colon carcinoma, leukemia, adrenocortical carcinoma, glioma, and prostate cancer (19–26). Treatment of tumor cells with Gos resulted in cell cycle arrest at G0/G1 phase, and one report suggested that Gos induced apoptotic cell death in leukemia cells, possibly through protein kinase C (PKC) pathway (27). However, studies on Gos-induced apoptosis are still limited and precise molecular circuitry of Gos-induced apoptosis, particularly in MM cells, remains elusive.

In the present study, we tried to elucidate the signaling pathways regulating Gos-induced apoptosis in MM cells. Besides a role as BH3 mimetics, we found that Gos inhibits interleukin (IL)-6 signaling, thereby dephosphorylates Bcl-2 and downregulates Mcl-1. This suggests that inhibition of IL-6 signaling may be an alternative mechanism for Gos-induced apoptosis in MM cells besides the interference of BH3-dependent interaction.

## Materials and methods

### Cells and cultures

Human myeloma cell line (OPM2) was obtained from the Japan Cancer Research Resources Bank (Tokyo, Japan). Cells were maintained in RPMI-1640 medium (Sigma, St. Louis, MO, USA) supplemented with 10% fetal bovine serum (FBS; Sigma), 100 U/ml penicillin, and 100 μg/ml streptomycin in a humidified atmosphere with 5% CO_2_. Cell morphology was examined by staining cytospin preparation of the cells with Giemsa solution. Viability of the cells was evaluated by trypan blue dye exclusion method.

### Reagents

Gossypol was purchased from Sigma and dissolved in DMSO at a stock concentration of 100 mM, which was stored at −30°C. The pan-caspase inhibitor Z-VAD-FMK, and JAK2 inhibitor AG490 were from Calbiochem (La Jolla, CA, USA).

### Antibodies

The antibodies for caspases-3, caspase-8, STAT3, pTyr705-STAT3, pSer727-STAT3, pSer70-bcl2, Bid, Bad, Akt, p38MAPK, pThr180/Tyr182-p38MAPK, cytochrome *c*, were from Cell Signaling Technology (Beverly, MA, USA). Antibodies for Bax and p21^CIP1^ were from MBL (Nagoya, Japan). Antibodies for β-actin, Rb, Mcl-1, bcl-2, bcl-xL, p-gp130, JAK2, Cyclin D2, cyclin E, CDK2, CDK4, PKCα, PP2A/Aα, PP2A/B56α, PP2A/C were from Santa Cruz Biotechnology (Santa Cruz, CA, USA). Antibodies for p-JAK2 or p27^kip1^ were from Upstate (Waltham, MA, USA) or BD Transduction Laboratories, respectively. Anti-ERK1/2 or p-ERK1/2 antibody was from Sigma. Secondary antibodies conjugated with horseradish peroxidase were obtained from GE Healthcare (Tokyo, Japan).

### Apoptosis assay

Apoptosis was examined by cellular morphology or staining cells with Annexin V-fluorescein isothiocyanate (FITC) and propidium iodide (PI) by Annexin V staining kit (BD Bioscience Pharmingen, San Diego, CA, USA) according to the manufacturer’s protocol. Stained cells were analyzed by FACSCalibur (Becton-Dickinson, San Jose, CA, USA) with CellQuest software (Becton-Dickinson).

For DNA fragmentation assay, cells were harvested and incubated in a lysis buffer [10 nM Tris-HCl (pH 7.4), 10 mM EDTA, 0.5% Triton 100-X] at 4°C, which were then centrifuged at 15,000 rpm for 15 min at 4°C. Supernatants were collected and incubated with RNase A (Sigma) at 50 μg/ml and proteinase K (Sigma) for 1 h at 37°C. DNA samples were subjected to 2% agarose gel and were visualized by ethidium bromide staining.

For pharmacological inhibition of apoptosis, cells were pre-incubated with 20 μM of pan-caspase inhibitor, Z-VAD-FMK for 2 h prior to addition of Gossypol (5 μM). The final concentration of DMSO in the experiment did not exceed 0.1%. Effect of Z-VAD-FMK was assessed by DNA fragmentation assay.

### Assay for mitochondrial transmembrane potential (MMP)

Cells were washed with PBS and subjected to staining with 40 nM of DioC6 (Sigma-Aldrich Japan, Tokyo, Japan) for 30 min at 37°C. The stained cells were washed with PBS and analyzed by flow cytometry.

### Cell cycle analysis

Cells were suspended in hypotonic solution [0.1% Triton X-100, 1 mM Tris-HCl (pH 8.0), 3.4 mM sodium citrate, 0.1 mM EDTA] and stained with 50 μg/ml of PI. Cells were analyzed by flow cytometry and the population of cells in each cell cycle phase was determined using ModiFIT software (Becton-Dickinson).

### Immunoblotting

Cells were lysed in a lysis buffer [1% NP40, 1 mM phenylmethylsulfonyl fluoride (PMSF), 40 mM Tris-HCl (pH 8.0) and 150 mM NaCl] at 4°C for 15 min. Mitochondria and cytosol were fractionated using the Mitochondria/Cytosol Fractionation kit (BioVision Inc., Mountain View, CA, USA). Cell lysates (15 μg of protein/lane) were fractionated in SDS-polyacrylamide gels and were transferred onto the nylon membranes (Immobilon-P; Millipore, Bedford, MA, USA). Membranes were probed with primary antibodies and horseradish peroxidase labeled secondary antibodies as described previously. Bound antibodies were detected by enhanced chemiluminescence (ECL) kit (Amersham, Buckinghamshire, UK).

### Statistical analysis

Data are expressed as mean ± standard deviation (SD) Statistical analyses were performed by unpaired Student’s t-test. P-values <0.05 were considered statistically significant.

## Results

### Gossypol induces apoptosis in multiple myeloma cells

**Figure 1:**
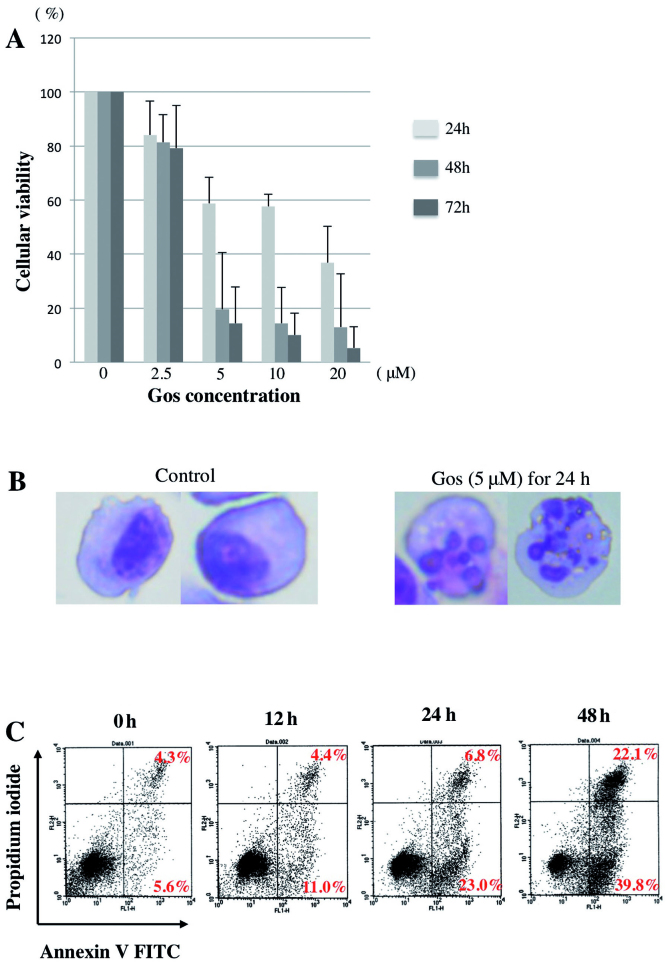
Growth inhibition and apoptosis induction of OPM2 cells by Gossypol. (A) OPM2 cells were treated with various concentrations (0–20 μM) of Gos for the time indicated (24–72 h). Viability of the cells was exmined by trypan blue dye exclusion method. Results were expressed as the mean ± SD (n=3). (B) Morphological changes of OPM2 cells by Gos-treatment. Cells were treated with 5 μM of Gos for 24 h, cytospun onto glass slides, and assessed for their morphology by Giemsa staining (original magnification, ×1,000). (C) Assessment of apoptosis by Annexin V/PI staining. OPM2 cells were treated with 5 μM of Gos for 0, 12, 24 or 48 h, and analyzed as described in Materials and methods. Representative figures from three independent experiments are shown.

We first examined the effect of Gos on the proliferation of MM cell line, OPM2 cells. As shown in Fig. 1A, Gos inhibited the proliferation of OPM2 cells in a dose- and time-dependent manner. Morphological examination revealed that treatment of OPM2 cells with 5 μM of Gos for 24 h clearly induced cell death with nuclear fragmentation, typical appearance of apoptosis (Fig. 1B). Furthermore, proportions of Annexin V^+^/PI^−^ as well as Annexin V^+^/PI^+^ cells were strikingly increased by 48-h treatment of Gos (Fig. 1C). Taken together, these results indicated that Gos induces apoptosis in OPM2 cells.

### Effect of Gos on cell cycle progression in myeloma cells

**Figure 2:**
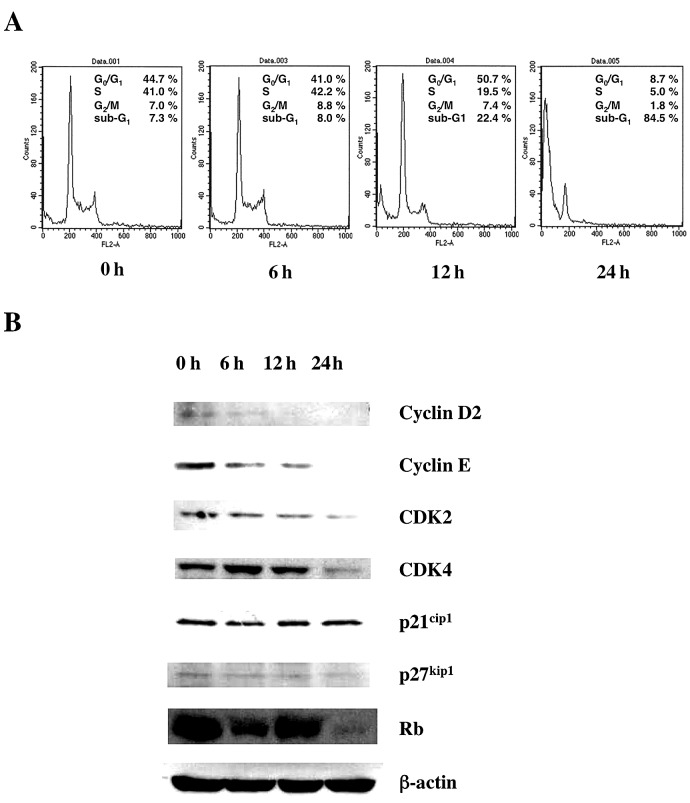
Cell cycle analyses of OPM2 cells treated with Gossypol. (A) Cell cycle analysis of OPM2 cells with Gos. Cell were cultured with 5 μM of Gos for 0–24 h and then stained with PI as described in Materials and methods. DNA content was analyzed by flow cytometry. Representative figures from three independent experiments are shown. (B) Expression of cell cycle related proteins in OPM2 cells treated with 5 μM of Gos for the indicated times. β-actin was used as a loading control.

Next we examined the effect of Gos on the cell cycle status of OPM2 cells. Cells were incubated with Gos and the cell cycle status was examined by flow cytometry at various time-points (Fig. 2A). The results demonstrated that Gos induced depletion of cells in S/G2/M phase and dramatic increase of cells in sub-G1 after 24 h of treatment. These results suggest that Gos induced cell cycle arrest at G1 followed by apoptosis in OPM2 cells.

To investigate the molecular mechanism of Gos-induced cell cycle arrest in OPM2 cells, the expression of cell cycle-associated genes was examined by western blotting. As shown in Fig. 2B, the expressions of Cyclin D2, Cyclin E, CDK2, CDK4 and Rb were decreased by Gos-treatment, whereas p21 and p27 were unchanged. These results indicate that Gos affected molecules driving cell cycle progression rather than inhibitors of cell cycle.

### Gossypol-induced apoptosis involves activation of caspase-3 and -8

**Figure 3:**
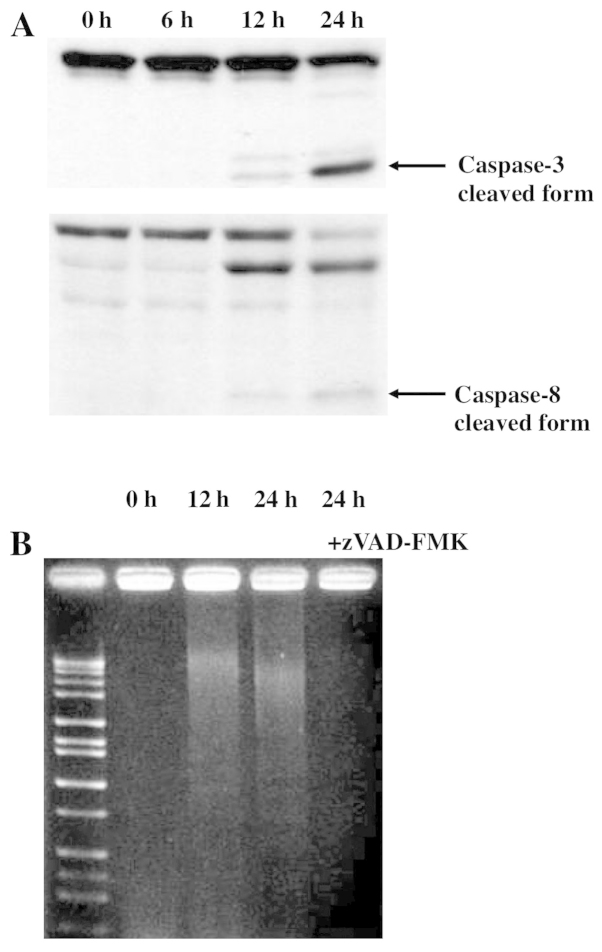
Effects of Gossypol on caspase activation. (A) Western blot analysis of caspase-3 and -8. OPM2 cells were treated with 5 μM of Gos for the indicated times. Activation of caspases as shown by the appearance of cleaved caspase-3 (upper panel) or caspase-8 (lower panel) is shown by the arrows. (B) DNA fragmentation assays. DNA extracted from OPM2 cells treated with 5 μM of Gos was subjected to agarose gel electrophoresis. DNA smear and ladder formation was observed from 12 h of Gos-treatment, which was effectively blocked by a pan-caspase inhibitor, Z-VAD-FMK (24 h + zVAD-FMK).

It is well known that the activation of caspases plays a pivotal role in the apoptosis-signaling pathway (28). We therefore examined the activation of caspases-3, a common effector caspase that integrates various death signals, during Gos-induced apoptosis in OPM2 cells. Active cleaved form of caspase-3 appeared from 12 h of Gos-treatment and became evident at 24 h (Fig. 3A). Furthermore, we found that Gos activated caspase-8, a mediator of death receptor-mediated apoptosis pathways, as shown by cleavage of the pro-form into the active form (Fig. 3A). The caspase activation was associated with fragmentation of DNA as shown by Fig. 3B. To further examine the role of caspases in Gos-induced apoptosis, we asked if caspase inhibitor could suppress the apoptotic processes in OPM2 cells. As expected, Gos-induced apoptosis in OPM2 cells as assessed by DNA fragmentation was completely inhibited by a pan-caspase inhibitor, Z-VAD-FMK (Fig. 3B). Of note, Z-VAD-FMK, when used alone, did not exert any effect on the proliferation of OPM2 cells (data not shown).

### Mitochondrial changes and decrease of Bid protein during Gos-induced apoptosis

**Figure 4:**
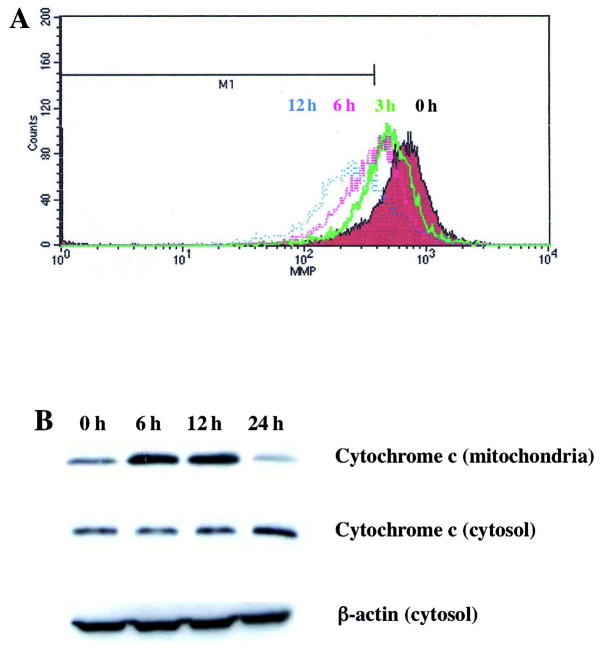
Gossypol activates mitochondrial dysfunction pathway of apoptosis. (A) Flow cytometric analysis of mitochondrial transmembrane potential (ΔΨ_m_). OPM2 cells were treated with 5 μM of Gos for 0–12 h, and mitochondrial membrane potential was measured by DioC6 fluorescence. (B) Release of cytochrome *c* proteins from mitochondria. Cytoplasmic or mitochondrial proteins were extracted from OPM2 cells treated with 5 μM of Gos for 0–24 h using Mitochondria/Cytosol Fractionation kit. β-actin was blotted as a loading control.

To examine the activation of mitochondrial apoptosis pathway by Gos, we next examined the mitochondrial changes evoked by Gos treatment. Mitochondrial changes during apoptosis include permeability transition pore opening and the collapse of the mitochondrial transmembrane potential (ΔΨ_m_), which results in the release of cytochrome *c* into the cytosol and subsequent activation of caspases. In order to examine these processes, we measured changes of mitochondrial ΔΨ_m_ in OPM2 cells treated with Gos by flow cytometry using DioC6. By treatment with 5 μM of Gos, ΔΨ_m_ of mitochondria decreased in a time-dependent manner in OPM2 cells (Fig. 4A). Furthermore, the decrease of ΔΨ_m_ was accompanied by the release of cytochrome *c* from mitochondria to cytosol (Fig. 4B). These results suggested that Gos-induced apoptosis is, at least in part, mediated through a mitochondria-dependent pathway.

**Figure 5:**
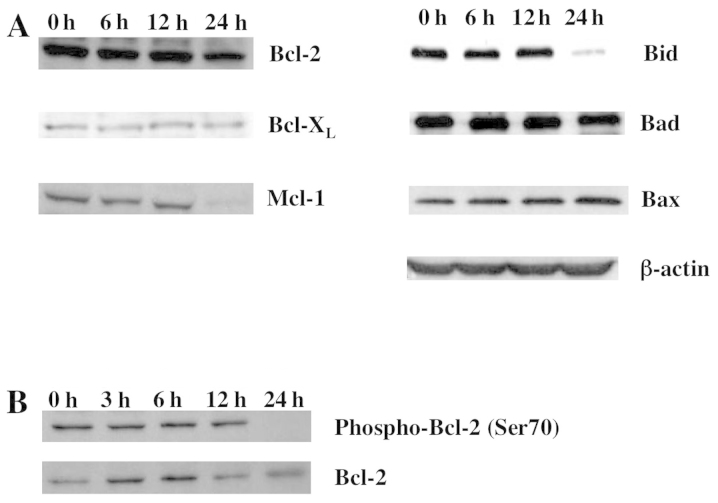
Expression of apoptosis associated proteins and phosphorylation of Bcl-2 during Gos-induced apoptosis. OPM2 cells were treated with 5 μM of Gos for 0–24 h and the expression of apoptosis associated proteins (A) or Ser-70 phosphorylation of Bcl-2 (B) was examined by western blot analysis. Blots are representative of three independent experiments.

We have also reconfirmed the activation of death receptor mediated pathways as shown by the activation of caspase-8 in Gos-induced apoptosis by checking the expression of Bid protein, since Bid is a substrate of activated caspase-8. As shown in Fig. 5A, Bid was clearly downregulated during the Gos-treatment. This result, together with the activation of caspase 8, indicated that the death receptor-mediated signaling pathway was also operational in the Gos-induced apoptosis in MM cells. Taken together, these results showed that Gos-induced apoptosis involved both mitochondria- and death receptor-dependent pathways.

### Expression of apoptosis-associated proteins in Gos-treated OPM2 cells

We next examined the expression of apoptosis-associated proteins during Gos-induced apoptosis in OPM2 cells. In addition to the decrease of Bid protein as shown above, MCL-1, one of the major anti-apoptotic proteins in hematopoietic cells, clearly decreased by 24 h of Gos-treatment. In contrast, however, Gos did not affect the levels of pro-apoptotic Bax and Bad protein as well as anti-apoptotic Bcl-2 and Bcl-X_L_ proteins (Fig. 5A).

Although protein levels did not change by Gos-treatment, we suspected that BCL-2 might be functionally impaired, since anti-apoptotic function of BCL-2 can be regulated by phosphorylation at serine 70 (Ser70) (29,30). In fact, Ser70 phosphorylation of BCL-2 was downregulated by 24 h of Gos-treatment (Fig. 5B), indicating that anti-apoptotic function of BCL-2 was perturbed by Gos-treatment. Taken together, these results suggest that downregulation of MCL1 and dephosphorylation of BCL-2 are responsible for Gos-induced apoptosis.

### Downregulation of ERK1/2 signaling by Gos may be responsible for BCL-2 dephosphorylation

**Figure 6:**
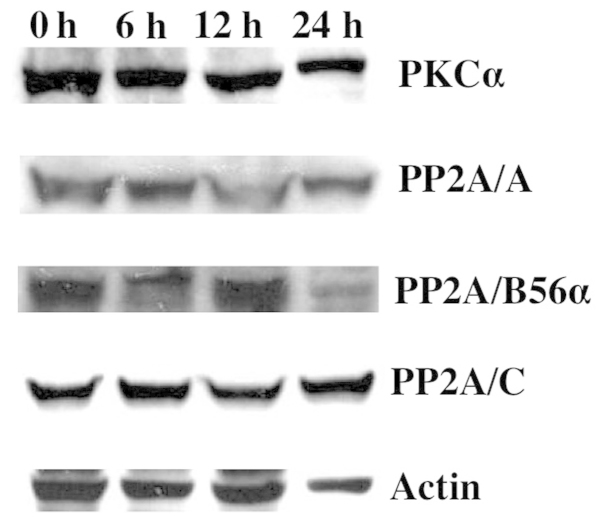
Expression of PKC and PP2A during Gos-induced apoptosis. OPM2 cells were treated with 5 μM of Gos for 0–24 h, and the protein levels of PKCα and three isoforms of PP2A (PP2A/A, PP2A/B56α and PP2A/C) was examined by western blot analysis. Blots are representative of three independent experiments.

**Figure 7:**
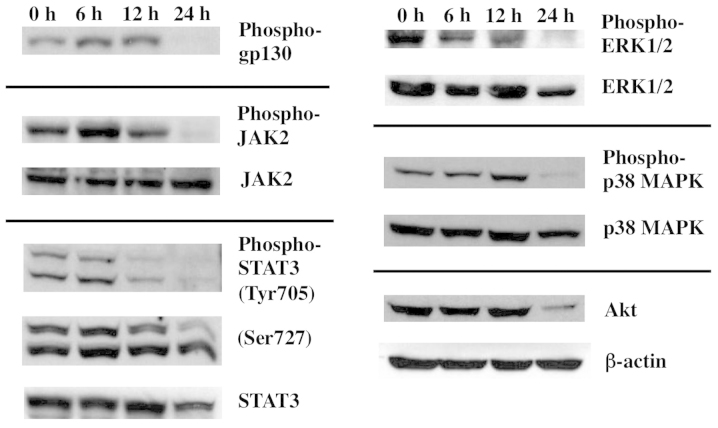
Gossypol inhibits IL-6 signaling. OPM2 cells were treated with 5 μM of Gos for 0–24 h, and the phosphorylation of signaling subunit of IL-6 receptor, gp130 and downstream signaling molecules, JAK2, STAT3, ERK1/2 and p38MAPK was examined by western blot analysis. Blots are representative of three independent experiments.

Recent studies have shown that ERK1/2, p38MAPK, protein kinase C (PKC) or protein phosphatase (PP) 2A were involved in the phosphorylation or dephosphorylation of BCL-2, respectively (31,32). To investigate the possible molecules regulating BCL-2 dephosphorylation during Gos-treatment, we first analyzed the expression levels of PKCα and PP2A in OPM2 cells. The results showed that protein levels of PKCα, PP2A/A, PP2A/B56α or PP2A/C did not change during 24 h of Gos-treatment (Fig. 6), suggesting that these enzymes did not affect the phosphorylation status of BCL-2 protein. In contrast, however, activation of ERK1/2 as examined by its phosphorylated form, was clearly decreased at 24 h of Gos-treatment (Fig. 7). Taken together, these results suggest that Gos induces dephosphorylation of BCL-2 through inhibition of ERK1/2.

### Inhibition of interleukin-6 signaling by Gos

It is well known that interleukin (IL)-6 plays a critical role in the proliferation of myeloma cells including OPM2 (33). To further gain insight into the upstream signaling pathways regulating Gos-induced apoptosis, we investigated the effect of Gos on the IL-6 signaling in OPM2 cells. IL-6 regulates cellular survival through three major signaling pathways: JAK2/STAT3, Ras/Raf/MEK/MAPK, and phosphoinositide-3 kinase (PI-3K)/Akt (34). Interestingly, treatment of OPM2 cells with Gos for 24 h inhibited phosphorylation of JAK2 and IL-6 signaling receptor, gp130, as well as the downstream signaling effectors such as STAT3, p38MAPK and ERK1/2 as mentioned earlier (Fig. 7). Downregulation of PI-3K/Akt pathway was also evident, since protein level of Akt itself decreased by Gos-treatment. These results suggest that Gos inhibits IL-6 signaling at a level between the ligand-receptor interaction and the activation of JAK2, which then leads to apoptosis in OPM2 cells.

### Inhibition of JAK2 results in dephosphorylation of BCL-2 and downregulation of MCL-1

**Figure 8:**
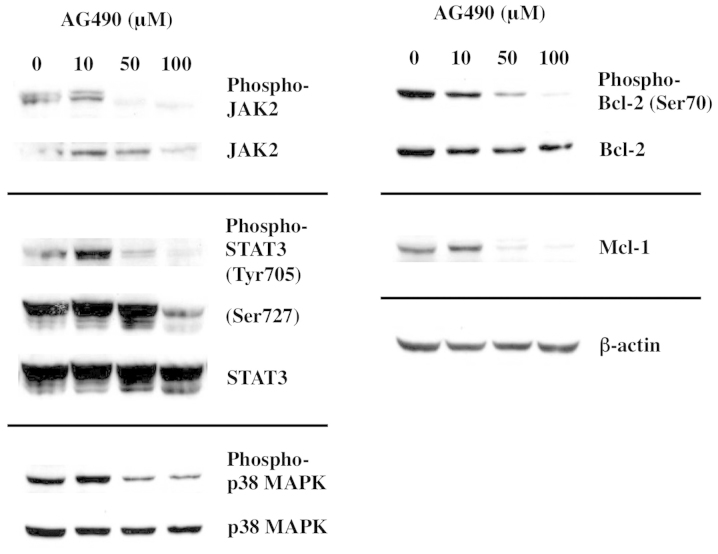
JAK2 inhibition mimics the effect of Gossypol on IL-6 signaling in OPM2 cells. OPM2 cells were treated with various concentrations of JAK2 inhibitor, AG490, for 24 h. Phosphorylation status and protein levels of JAK2, STAT3, p38MAPK, Bcl-2 or Mcl-1 were examined by western blot analysis. Results are representative of three independent experiments.

To elucidate the acting point of Gos-mediated inhibition of IL-6 signaling, we examined whether inhibition of JAK2 was sufficient to cause deregulation of apoptosis-associated proteins evoked by Gos stimulation. As shown in Fig. 8, treatment of OPM2 cells with JAK2 inhibitor AG490 inhibited the phosphorylation of JAK2 as well as the downstream effectors such as STAT3 and p38MAPK. As expected, dephosphorylation of BCL-2 and decrease of MCL-1 were induced by AG490 treatment in parallel to JAK2 inhibition.

These data suggest that Gos-induced deregulation of Bcl-2 and Mcl-1 was mediated through inhibition of IL-6 signaling at the level leading to JAK2 activation.

## Discussion

Gos is a natural small molecule inhibitor for Bcl-2 or Bcl-X_L_ that has been shown to act as a potent inducer of apoptosis in multiple tumor cell types. Previous studies have shown that Gos downregulated the expression of Bcl-2/Bcl-X_L_/Mcl-1 proteins in multiple tumor cell lines. In contrast, however, our data indicated that Gos treatment did not affect the protein levels of Bcl-2 or Bcl-X_L_. Instead, Gos attenuated Ser-70 phosphorylation of Bcl-2, which is critical for executing its full and potent anti-apoptotic effects (29,30). It has been reported that Ser70 of Bcl-2 was phosphorylated by ERK1/2 kinases (29,35), and we demonstrated that activated form of ERK1/2 (phospho-ERK1/2) was in fact downregulated by Gos treatment. We went on to show that Gos also downregulated phosphorylated forms of JAK2, gp130, and STAT3, critical components of active IL-6 signaling. Taken together, these results indicate that Gos induces dephosphorylation of Bcl-2 at Ser-70 through global inhibition of IL-6 signaling.

We also demonstrated that another anti-apoptotic protein, Mcl-1, was downregulated by Gos treatment. This should be due to the inhibition of IL-6 signaling by Gos as well, since Mcl-1 is a downstream target of IL-6 (36,37). Mcl-1 is frequently overexpressed in MM cells (38), and has been shown to be critical for their survival (39). Some reports even demonstrated that Mcl-1, rather than Bcl-2 or Bcl-X_L_, plays a primary role in the survival of MM cells (40). Therefore, downregulation of Mcl-1 is definitely one of the major pathways of Gos-induced apoptosis.

Gos shares a structural profile of BH3 mimetics with other inhibitors for Bcl-2/Bcl-X_L_ such as Genasense, TW-37, Obatoclax or ABT-263 that are presently under clinical trials (10,28). It has been therefore recognized that displacement of BH3-only proteins from Bcl-2/Bcl-X_L_ or direct activation of BAX or BAD through BH3-mediated interaction is a major molecular mechanism for Gos-induced apoptosis in cancer cells (10). However, as described above, we demonstrated that Gos inhibits IL-6 signaling in MM cell line and induces apoptosis through downregulation of Mcl-1 and Bcl-2 dephosphorylation. These results propose inhibition of IL-6 signaling as a novel mechanism for Gos-induced apoptosis in MM cells.

Molecular mechanism of Gos-mediated inhibition of IL-6 signaling is not clear at present. In cytokine signaling, ligand-induced dimerization of receptor chains provokes activation of Jak kinases, which then leads to phosphorylation of receptor chains and initiates downstream signaling cascades (41,42). In this study, we demonstrated that Gos inhibited phosphorylation of JAK2 as well as major downstream molecules of IL-6 signaling such as STAT3 and ERK1/2. These results suggest that inhibition of JAK2 is the primary effect of Gos on IL-6 signaling in MM cells. In support of this notion, pharmacological inhibition of JAK2 in OPM2 cells showed the biochemical effects highly similar to Gos-treatment, such as dephosphorylation of Bcl-2 and decrease of Mcl-1. Inhibition of JAK2 activation by Gos could be occurring at the level of ligand binding, receptor dimerization, or JAK2 itself. Elucidating precise molecular mechanism how Gos inhibits JAK2 activation requires further study.

During the preparation of this report, we noticed that another group reported apoptosis inducing effect of Gos in MM cells (43). In contrast to our findings, they showed that Gos decreased protein levels of Bcl-2 and Bcl-X_L_ by flow cytometry, although they did not examine the phosphorylation status of Bcl-2. One possible reason for this discrepancy could be the different concentrations of Gos used in either study. They used Gos at 25 μM, while we took 5 μM in most experiments. In fact, we observed moderate decrease of Bcl-2 protein when cells were treated with Gos at 10 μM or higher for 24 h (data not shown). However, our data clearly indicate that Gos-induced apoptosis occurs at 5 μM, a concentration that readily induces Bcl-2 dephosphorylation (Figs. 1–4). This strongly suggests that decrease of Bcl-2/Bcl-X_L_ protein levels is not the primary cause for apoptosis induction by Gos in MM cells.

In conclusion, we demonstrated that Gos, a natural BH3 mimetics, induces apoptosis in MM cells not only through displacement of BH3-only proteins from Bcl-2/Bcl-X_L_, but also via the inhibition of IL-6 signaling. Future study will focus on the mechanism how Gos inhibits IL-6 signaling in MM cells. It is expected that this allows us to obtain vital information on novel strategies for IL-6 inhibition, which leads to a development of new drugs for MM treatment.
